# PEO Coatings Modified with Halloysite Nanotubes: Composition, Properties, and Release Performance

**DOI:** 10.3390/ijms24010305

**Published:** 2022-12-24

**Authors:** Igor Imshinetskiy, Victoria Kashepa, Konstantine Nadaraia, Dmitry Mashtalyar, Sergey Suchkov, Pavel Zadorozhny, Aleksander Ustinov, Sergey Sinebryukhov, Sergey Gnedenkov

**Affiliations:** Institute of Chemistry, Far Eastern Branch, Russian Academy of Sciences, Vladivostok 690022, Russia

**Keywords:** plasma electrolytic oxidation, halloysite nanotubes, multifunctional coatings, caffeine release

## Abstract

In this work, the properties of the coatings formed on the Mg-Mn-Ce alloy by plasma electrolytic oxidation (PEO) in electrolytes containing halloysite nanotubes (HNTs) were investigated. The incorporation of halloysite nanotubes into the PEO coatings improved their mechanical characteristics, increased thickness, and corrosion resistance. The studied layers reduced corrosion current density by more than two times in comparison with the base PEO layer without HNTs (from 1.1 × 10^−7^ A/cm^2^ to 4.9 × 10^−8^ A/cm^2^). The presence of halloysite nanotubes and products of their dihydroxylation that were formed under the PEO conditions had a positive impact on the microhardness of the obtained layers (this parameter increased from 4.5 ± 0.4 GPa to 7.3 ± 0.5 GPa). In comparison with the base PEO layer, coatings containing halloysite nanotubes exhibited sustained release and higher adsorption capacity regarding caffeine.

## 1. Introduction

Most modern engineering areas face a demand for lightweight construction materials. Magnesium alloys combine high performance regarding mechanical properties with low density, which explains their wide application in the design of car engine blocks, gearboxes, steering columns, rotorcraft control-system levers, plane light-weight structure frames, and other machinery parts with a weight-sensitive application area [1,2,3,4,5]. 

Beyond the industrial sector, biomedical engineering considers the potential use of magnesium alloys for lightweight biodegradable orthopedic implants that eliminate the necessity of a second surgical procedure for implant removal after bone healing [6,7,8,9]. The load-bearing capacity, harmless products of degradation [8], and the essential role of Mg in the human metabolism [10] make this metal and some of its alloys prospective biomaterials. 

However, the high susceptibility of magnesium alloys to corrosion and their poor wear resistance inhibit their application in moist and adverse conditions as well as implant material. These issues can be effectively alleviated by the surface engineering techniques aimed at creating protective coatings with various functional properties [11,12,13]. One of the most promising methods of surface modification is plasma electrolytic oxidation (PEO) [14], which induces the formation of thick oxide layers on the surfaces of the valve metals under supercritical conditions [15,16]. PEO became widely investigated due to the multifunctional performance of the formed coatings and the low requirement for the treated surface quality. Depending on the content of the obtained layer, the protective properties of the PEO coatings can be accompanied by photocatalytic activity [17,18], antibacterial properties [19,20], improved hardness [21], etc. The possibility of managing coatings properties through a wide range of adjustable process parameters [22] enables the formation of PEO layers for various fields, including mechanical [23,24] and biomedical engineering [13,25]. 

The PEO technique allows the formation coatings of bioactive composition with a convoluted morphology, providing both the biocompatibility [26,27,28] and osteointergation [29,30,31] of a modified surface, which makes PEO coatings formed on magnesium alloys an appropriate basis for biodegradable implant design. The implant setting could be assisted by the controllable release of pharmaceuticals from a modified PEO layer that, depending on the substance used, would prevent implant-associated infections and inflammatory processes, and accelerate bone tissue recovery. 

Halloysite nanotubes (HNTs) can serve as an entrapment system in PEO layers for the loading, storage, and controlled release of hydrophilic and lipophilic [32,33,34] active molecules, such as drugs [35,36,37,38], vitamins [39], proteins [40,41,42], and corrosion inhibitors [43,44]. The HNTs’ incorporation mechanism into the PEO coating during the oxidation process conforms to the mechanism described for other nanoparticles [45,46]. However, there is still no clear understanding of the destructive impact of plasma discharges on the encapsulating properties of the nanocontainers, including HNTs. 

HNTs are relatively inexpensive and accessible nanoparticles of natural origin; their deposits are quite common for lake sites in Australia, New Zealand, the USA, and Russia [47]. HNTs present the chemical formula of Al_2_Si_2_O_5_(OH)_4_•nH_2_O, the outer surface of the nanotubes is formed by siloxane groups (Si-O-Si), while the inner surface consists of aluminol groups (Al-OH) [48,49]. The encapsulating ability of the HNTs is mostly attributable to the interaction of their lumen aluminol groups with guest molecules by hydrogen bonding, whose strength increases with the dipole moment of the loaded substances [32,50,51]. 

Many papers have been devoted to the adsorption properties of HNTs [42,52,53]; however, information on the preservation of the adsorption capacity of HNTs embedded in PEO coatings, as well as on the possibility of single-step modification of PEO layers by HNTs loaded with active molecules, is still insufficient. The key issues that require investigation are the oxidizing effect of PEO on organic substances and its destructive effect on HNTs.

Release tests of the samples without HNTs and containing pre-loaded and raw HNTs allow evaluation of the preservation of the adsorptive properties of the HNTs and guest molecules in their lumen throughout the PEO process, as well as the relationship between the surface adsorption of the obtained coatings and the presence of HNTs.

The development of corrosion-resistant coatings modified by nanocontainers on magnesium alloys meets the current needs of modern science, medicine, and technology in lightweight products robust to different operating conditions and environments. The determination of the influence of strong electrical fields, extreme temperatures, and pressures realized during the PEO process on the physical and chemical behavior of incorporated HNTs has great scientific and practical significance for the further development of the theoretical framework behind multifunctional PEO coatings. 

## 2. Results and Discussion

### 2.1. Morphology and Composition of the Coatings

**Figure 1:**
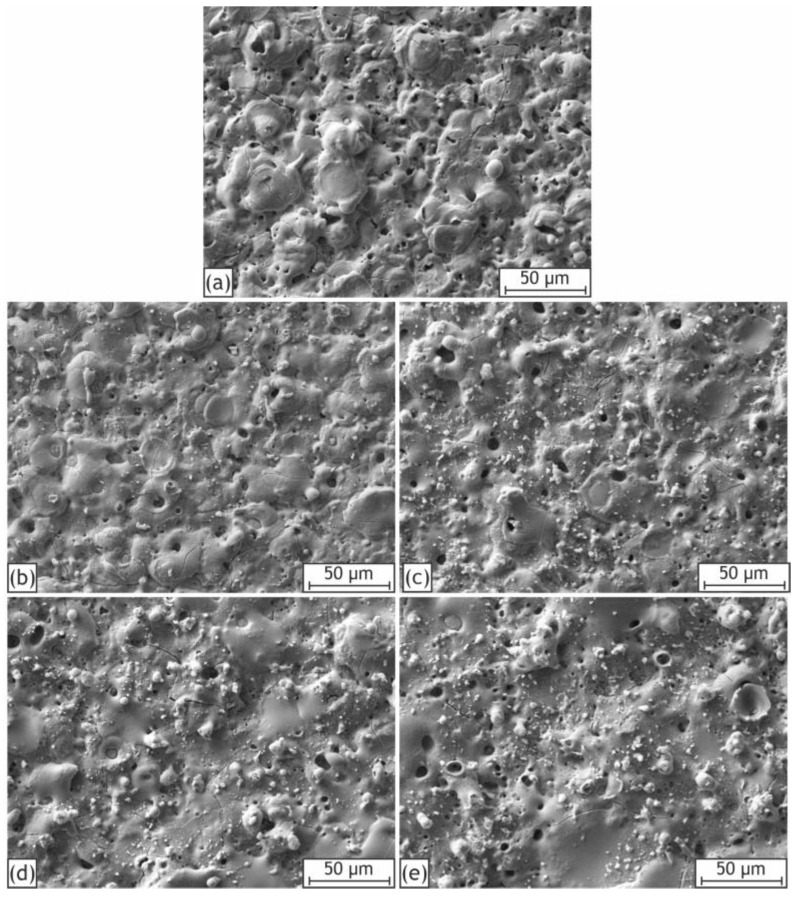
SEM images of the coatings surfaces: H0 (**a**), H10 (**b**), H20 (**c**), H30 (**d**), H40 (**e**).

The SEM images of the samples’ surfaces are presented in Figure 1. The surface morphology of the obtained samples is typical for PEO coatings with volcano-like or crater-like surface structures [15,54,55]. The addition of HNTs to the electrolyte elicited the formation of a more rugged and irregular surface compared to the base PEO coating. The clusters and agglomerates of the nanoparticles can be observed for the H10, H20, H30, and H40 samples. 

**Figure 2:**
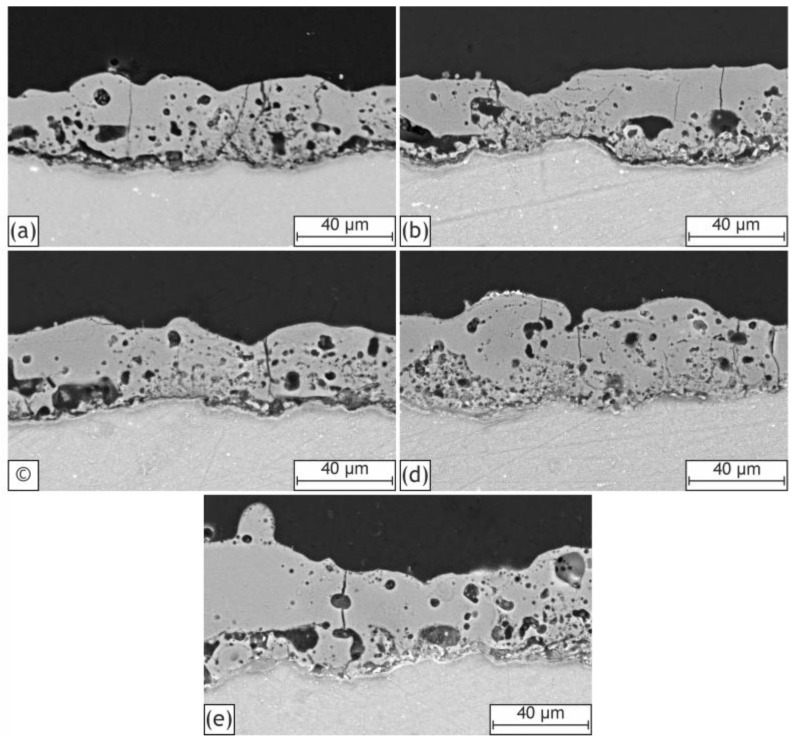
SEM images of coatings cross sections: H0 (**a**), H10 (**b**), H20 (**c**), H30 (**d**), H40 (**e**).

The SEM images of the coatings cross sections are shown in Figure 2. The HNTs strongly affect the internal porosity and thickness of the formed layers. It may be seen that the thickness increases gradually from H0 up to H40 (Table 1), which can be explained by the influence of nanoparticles’ addition on the electrical response of the system. Porosity and thickness parameters are closely interrelated, and both depend on the kinetics of the PEO process. The sintering of the HNTs contributes to the creation of layers with a more heterogeneous and porous morphology, affecting the energy and duration of the further plasma discharges. As a result, the porosity of the oxide layers grows proportionally to the HNTs concentration (Table 1). At the same time, porous coatings undergo frequent breakdowns in the weak dielectric points [15,54,55,56,57], which leads to the formation of more thick and less dense coatings. 

**Figure 3:**
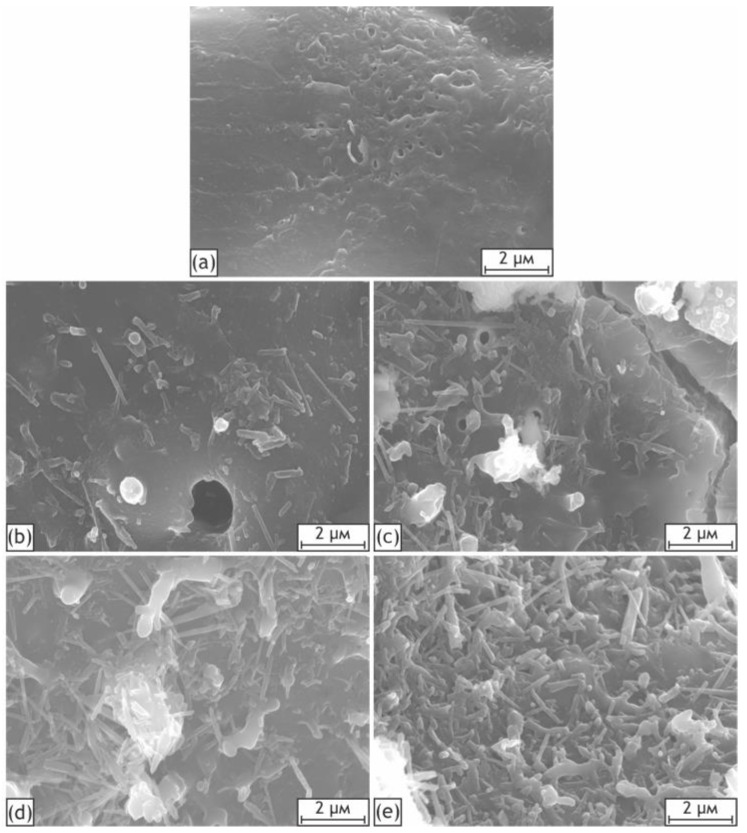
High-magnification SEM images of coatings surface: H0 (**a**), H10 (**b**), H20 (**c**), H30 (**d**), H40 (**e**).

As can be seen from the high-magnification SEM images of the samples’ surfaces (Figure 3), the occurrence of irregularities increases with the concentration of nanoparticles in the electrolyte. Large clusters of nanoparticles become clearly distinguishable for H20-H40 samples. Individual HNTs can be observed for all samples containing nanoparticles, while the base PEO coating possesses a much smoother appearance.

**Figure 4:**
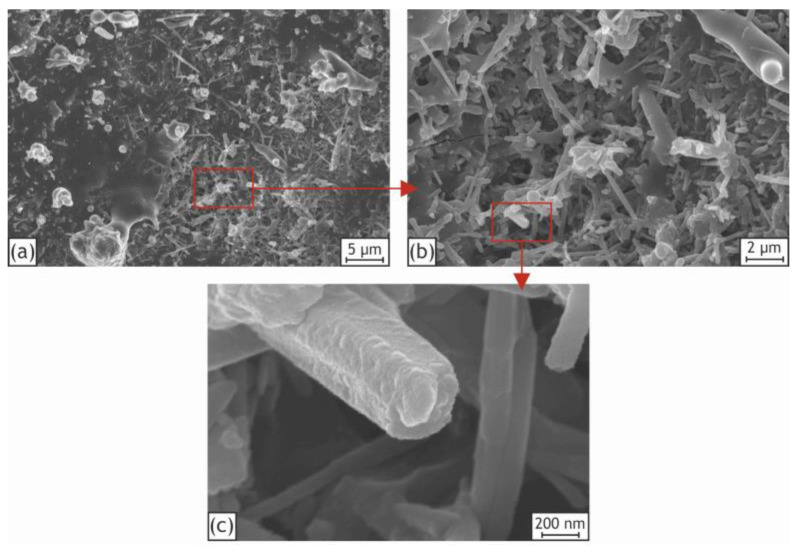
High-magnification SEM images of H40 sample (**a**) with details of the nanoparticles agglomerates (**b**) and HNTs themselves (**c**).

More detailed SEM images of the incorporated nanoparticles and agglomerates on the surface of the H40 sample are shown in Figure 4. The red frames within the pictures represent areas that were magnified and analyzed. The incorporated particles retain their characteristic tubular shape under the strong conditions of the PEO process, which positively affects the adsorptive capacity of the HNTs and the formed layers. 

**Figure 5:**
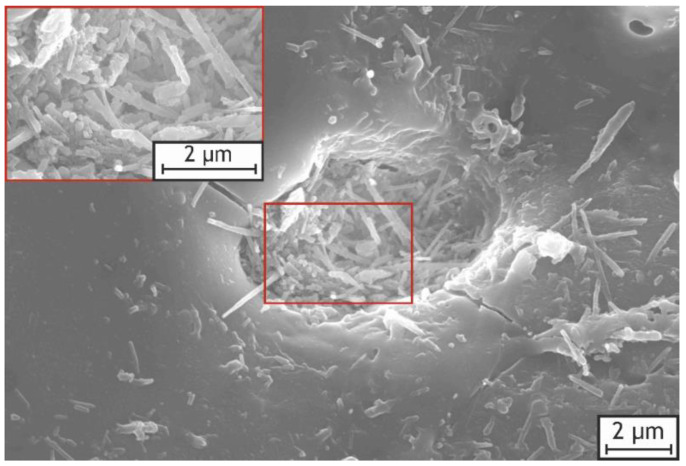
High-magnification SEM images of the H10 sample pore.

Based on the obtained results, it can be proposed that two main mechanisms of the HNTs’ incorporation take place. One part of HNTs is adhered to the bottom of the pores by electrophoretic force and sintered directly throughout the plasma discharge treatment, while the other part of HNTs, from the electrolyte in affinity to the substrate surface, is seized mechanically by the molten oxide layer. Moreover, as can be seen from the SEM images of the pore (Figure 5), part of the HNTs deposited onto the pore bottom maintained their original tubular structure as well. The nanoparticles are present at the bottom of the pores and discharge channels due to both the incorporation mechanism and the high surface energy of these locations mentioned by other scholars [46,58].

**Figure 6:**
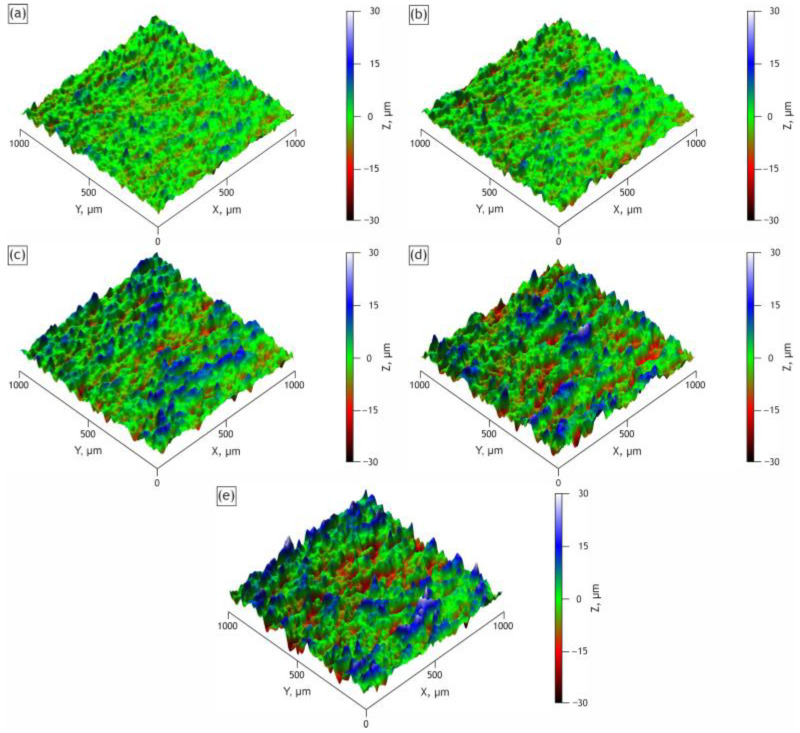
Surface topography of the H0 (**a**), H10 (**b**), H20 (**c**), H30 (**d**), H40 (**e**) samples.

The results of surface topography, thickness, and porosity measurements are presented in Table 1. The increasing roughness parameter over the samples indicates HNTs introduction. As can be seen from the 3D surface maps, the roughness increases steadily from the H0 to the H40 sample (Figure 6). 

The thickness of the layers is directly proportional to the HNTs concentration in the electrolyte. The H40 sample has a coating with an utmost thickness of 62 ± 8 µm, which is 1.2 times higher than the value obtained for the base PEO coating. 

**Figure 7:**
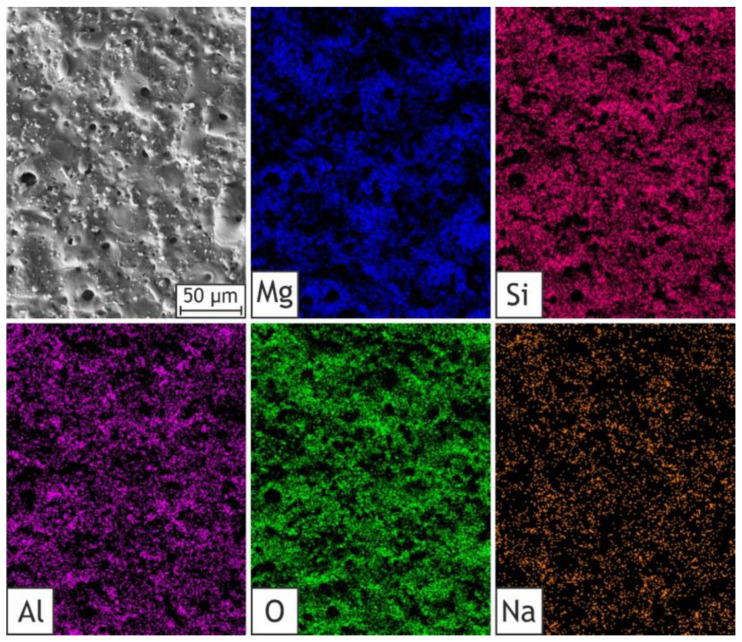
SEM image and EDS maps of elements distribution for the H40 sample: Mg, Si, Al, O, Na.

Element distribution maps obtained by EDX confirm aluminum presence on the coating surface (Figure 7). In view of the fact that this element is a component of the HNTs only (base PEO layer does not contain Al), its presence corresponds with nanoparticles incorporation. The presence of silicon indicates both the incorporation of the nanoparticles and substrate reaction with silicate ions during the PEO treatment. Oxygen is assigned to such components of the formed layers as oxides, silicates, and HNTs. The presence of sodium is attributable to the cation’s sorption from the electrolyte on the coating’s surface. The presence of magnesium is caused by the substrate oxidation into MgO, Mg_2_SiO_4_, and other derivatives [55].

**Figure 8:**
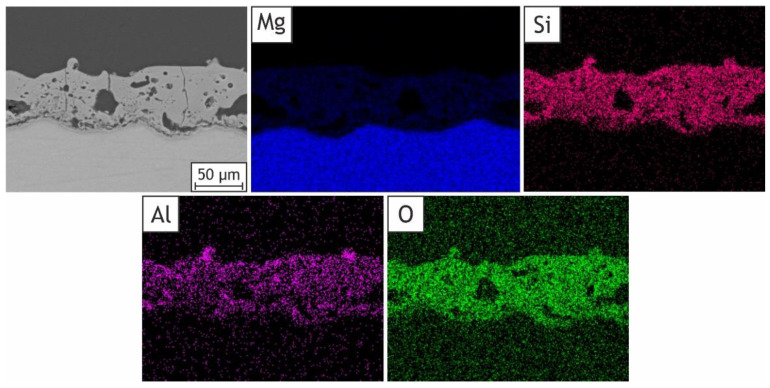
SEM image and EDS maps of elements distribution for the cross section of H40 sample: Mg, Si, Al, O.

The discrepancy in aluminum concentration between the outer layer and inner one can be seen on the cross-sectional element distribution map: its presence is higher on the surface of the sample in comparison with the internal layer (Figure 8). In particular, clusters with a high aluminum content can be observed on the surface of the H40 sample. This can be explained by the outward and inward coating growth that was proved in different research works [54,55]. Even though the first stage of the coating development is accompanied by the inclusion of nanoparticles [58], the access of halloysite nanotubes to the inner layer of the coating on the further stages of inward coating growth is limited by the size of the particles. On the contrary, HNTs readily reach and incorporate into the forming outer layer of the PEO coating, which leads to the difference in Al presence between the outer and inner layers of coating. 

The HNTs incorporation was also confirmed by the X-ray fluorescence spectrometry results (Table 2). The increase in aluminum concentration on the surface of the studied samples is obvious for all coatings obtained in the electrolyte containing HNTs, which conform to the previously obtained results.

The X-ray diffraction data of the used nanomaterial are represented in Figure S1. Considering the specificity of the XRD method and X-ray penetration depth, the diffractograms obtained for the PEO samples have no discernible peaks corresponding to halloysite. The intense peaks of a magnesium substrate overlap with halloysite peaks due to a low relative content of the nanoparticles in the samples. Therefore, diffractograms for the obtained samples are not presented. 

**Figure 9:**
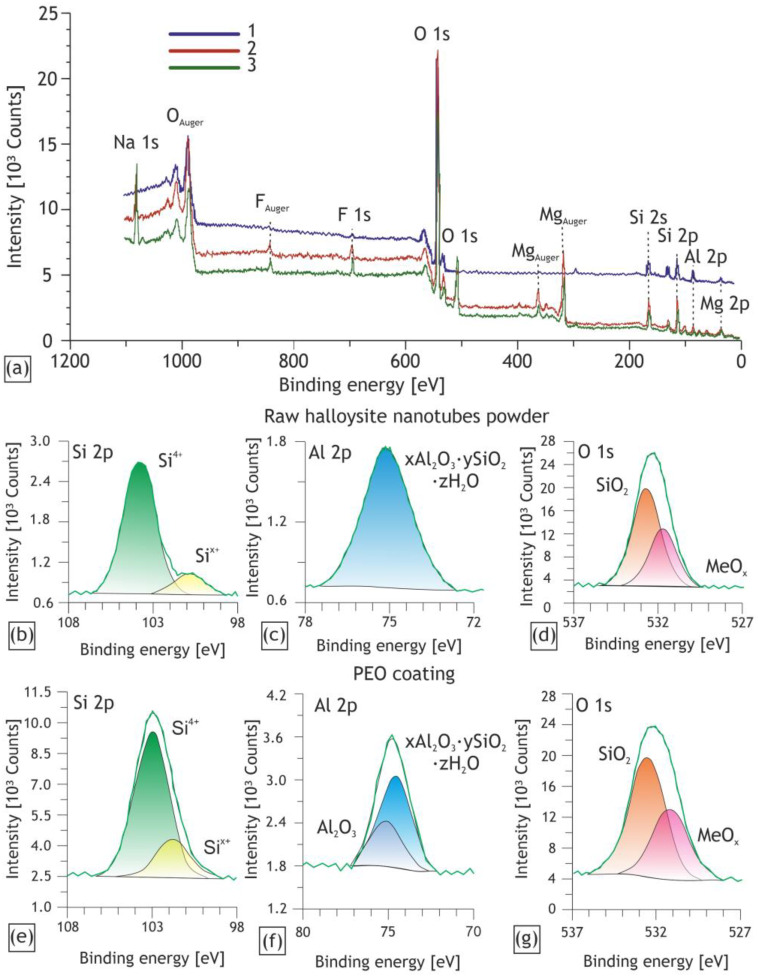
XPS survey spectra of the surface of the H40 coating: spectrum for raw HNTs powder (1), as-prepared H40 sample (2) and for the sample after 5 min Ar^+^ etching (3) (**a**); high-resolution XPS spectra of O 1s (**b**), Si 2p (**c**), Al 2p (**d**) for the raw HNTs and O1s (**e**), Si 2p (**f**), Al 2p (**g**) for the H40 sample.

Figure 9 illustrates the XPS survey and high-resolution spectra of the HNTs and PEO coating obtained in the electrolyte containing 40 g/L of the HNTs. In the represented survey spectra, binding energies associated with the elements were observed previously in element maps and discussed in the EDX analysis results. The acute reduction in the sodium and fluorine content after the etching can be observed, which confirms adsorption of the water-soluble electrolyte components on the coatings surface (Table 3). While Na content is completely dependent on the surface adsorption capacity and decreases substantially after the etching, part of the F^−^ ions react with the substrate with conversion into MgF_2_ during the PEO process [55,59], and therefore, the fluorine concentration changes to a lesser extent.

According to the calculated data, oxygen forms non-metal and metal oxides. As it can be seen from the high-resolution spectra, oxygen remained in the same states in both the raw powder of HNTs and the obtained coatings. 

According to the deconvolution of the XPS spectra, the silicon was found bound in two forms: in the common 4+ oxidation state and in a less oxidized state (peak of E_b_ about 101 eV), which presumably separates quartz and products of chemical or plasma–chemical reactions from the Si in its aluminosilicate state [60,61]. The spectrum presented in Figure 9a is distinguished from the spectrum shown in Figure 9e with a more distinct peak in the lower energy region. These changes in the intensity are attributable to the components of the electrolyte, namely metasilicate. The magnesium substrate reacts with silicate ions during the PEO process and forms forsterite with Si^4+^ [62,63]. Accordingly, the peak of this silicon state is more intense for the PEO coating compared to the raw material.

The Al 2p peak of the obtained PEO coating can be deconvoluted into two peaks, which indicates the presence of aluminum in two chemical states. The predominant peak in the lower energy region presumably corresponds to aluminum, which is part of the aluminosilicate (halloysite) [64,65]. The higher energy component presumably corresponds to the dehydroxylation product of the HNTs: Al_2_O_3_. Since the plasma discharge temperature in the PEO process reaches about 4500–10,000 K [56], the reactive incorporation of halloysite nanotubes, accompanied by their thermochemical transformations, is taking place. The HNTs conversion involves the segregation of aluminum oxide, which was mentioned in the work of Kissinger [66] and some other recent papers [67,68]. It is worth noting that the higher-energy component is absent in the spectrum of the raw nanoparticles, which confirms the assumption of the dehydroxylation of the HNTs under PEO. The formation of the secondary phase of Al_2_O_3_ is of particular interest, as it factors in the wear and corrosion resistance of the formed coatings. 

### 2.2. Electrochemical Properties of the Coatings

**Figure 10:**
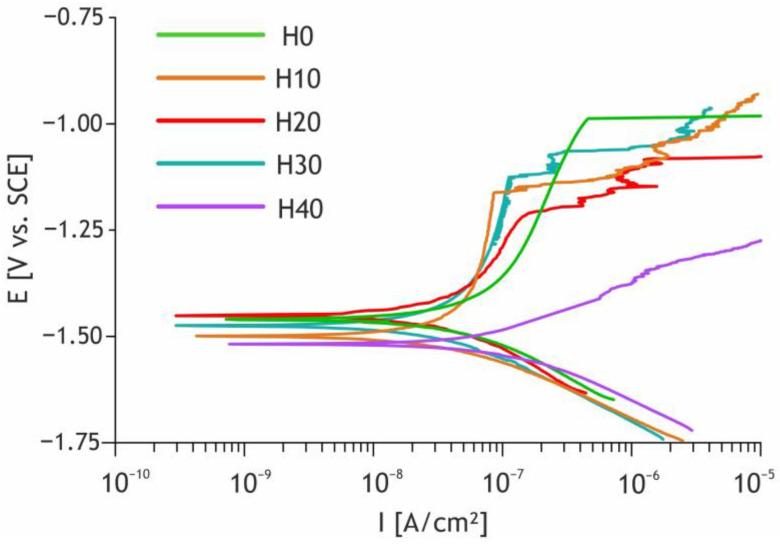
Polarization curves for the studied samples.

To provide a deeper insight into the influence of nanoparticles incorporation on the characteristics of the coatings, the electrochemical performance of the obtained samples was assessed by electrochemical impedance spectroscopy and potentiodynamic polarization techniques. The change in corrosion properties of coatings after the HNTs incorporation is evident from the analysis of the polarization curves represented in Figure 10 and the calculated performance specified in Table 4.

All samples containing nanoparticles, except H40 sample, demonstrated a decrease in the corrosion current density in comparison with the base PEO layer. The highest corrosion resistance was demonstrated by the H20 sample; it showed i_corr_ being more than two times lower than the value observed for the base PEO coating (from 1.1 × 10^−7^ to 4.9 × 10^−8^ A/cm^2^). Additionally, a distinct increase in the polarization resistance for the H10, H20, H30 samples in 1.3–1.8 times compared to the samples obtained in the electrolytes without nanoparticles could be observed. The H20 sample exhibited the highest polarization resistance of 1.2 × 10^6^ Ω∙cm^2^, which is almost two times higher than the R_p_ value for the H0 sample.

These characteristics can be explained by the incorporation of the HNTs, which led to partial pore sealing with the chemically stable HNTs and products of their thermal conversion. As was already noted for the results of the XPS test of the obtained coatings, they presumably include quartz and aluminum oxide, which contribute to the electrochemical behavior of the coatings. Moreover, the detailed SEM images of the pore demonstrated sintering of the HNTs to the bottoms of the pores and the infilling of incompletely closed channels with sintering products (Figure 5). Thus, HNTs seal the surface defects of the coatings and improve their chemical stability.

Once the concentration of the HNTs in the electrolyte reaches a value of 30 g/L, the anticorrosive properties of the formed coatings start to deteriorate. This tendency can be explained by an increase in the heterogeneity and porosity of the coatings, which result in the penetration of the aggressive environment toward the magnesium substrate through the defects and increase corrosion current density.

**Figure 11:**
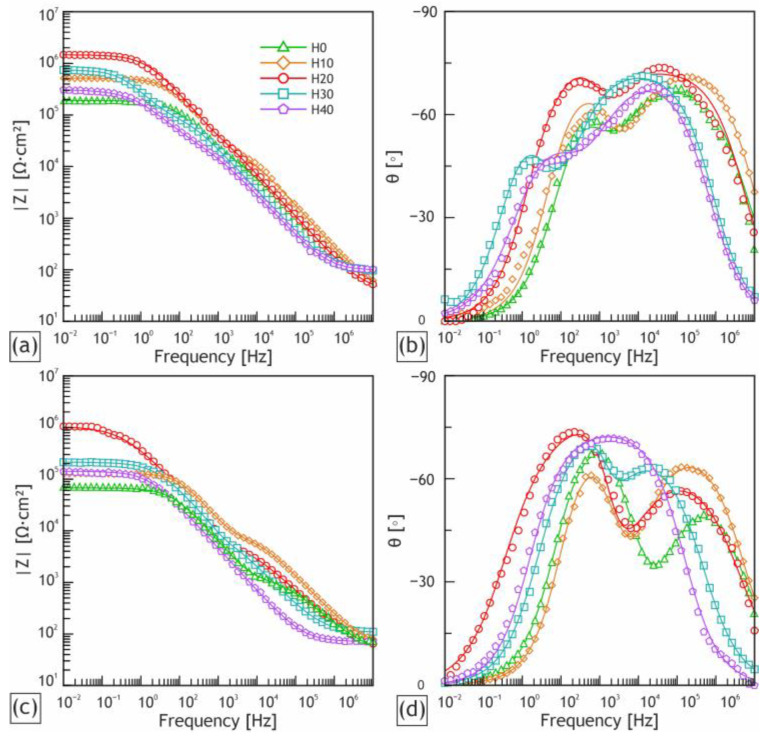
Bode plots (dependences of impedance modulus |Z| and phase angle θ on frequency for the obtained samples. Spectra were acquired after exposure to the corrosive medium for 2 h (**a**,**b**) and 24 h (**c**,**d**). Impedance spectra presented by experimental data (scatter plot) and fitting curves (solid lines).

**Figure 12:**
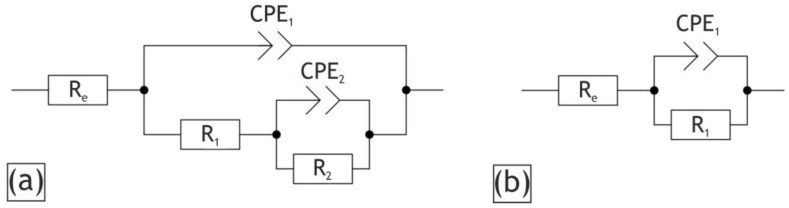
Equivalent electrical circuits used for fitting the impedance spectra.

The experimental data obtained by EIS are presented in the Bode plots (Figure 11) as dependencies of the impedance modulus (|Z|) and phase angle (θ) on frequency (f). For the impedance spectra fitting the appropriate equivalent, electric circuits (EECs) were used. The Bode plots have two low- and high-frequency bends (Figure 11b,d), which are responsible for the capacitance of the whole coating (CPE_1_) and the resistance of the porous sublayer (R_1_) and the capacitance and resistance of the non-porous sublayer (CPE_2_ and R_2_), R_e_ is a resistance of the electrolyte. Therefore, these two time constants presented in experimental spectra are accountable for the two different sublayers, which can be modelled with two series-parallel R-CPE-chains (Figure 12a). It should be noted that the behavior of the H40 sample after 24 h exposure to corrosive media can be modelled by the EEC with the one time constant presented in Figure 12b, where the single R-CPE-chain is responsible for charge transfer through the PEO layer with high porosity.

The constant phase element (CPE) was used in this work instead of the capacitance because of the heterogeneity of the coating sublayers. The impedance of the CPE is calculated in accordance with Equation (4):

(1) $$Z_{\mathrm{CPE}}=1∕Q{\left(\dot{J}\omega \right)}^{n},$$

where Q is the frequency independent parameter, $\dot{J}$ is the imaginary unit, ω is the angular frequency and n is the exponential coefficient. 

The results of the fitting of the experimental impedance spectra are presented in Table 5. The R_e_ value according to calculations was constant for all the studied samples and approximately equal to 30 Ω × cm^2^.

All samples with incorporated HNTs showed a higher impedance modulus at the lowest frequency compared to the base PEO coating. In the set of the samples the increase in the |Z|_f = 0.01 Hz_ can be observed up until the H30 sample, then the impedance modulus begins to decrease. The H20 sample showed the highest value of |Z|_f = 0.01 Hz_ (1.26 × 10^6^ Ω × cm^2^) after 2 h of exposure, which is 10 times higher than the value obtained for the base PEO coating (1.21 × 10^5^ Ω × cm^2^). 

The increase in the R_1_ values, especially for the H20 sample, shows the growth of the porous sublayer resistivity as a consequence of the incorporation of HNTs, as was indicated previously for the SEM images and potentiodynamic polarization tests. The R_1_ value for the H20 sample is 4 times higher compared to the one for the sample with the base PEO coating (due to more narrow pores), while the R_2_ value is 8 times higher for the same matter. This behavior points to the remarkably thicker nonporous sublayer of the H20 sample among other samples, which determines its high anticorrosive properties.

The Q_1_ values significantly contribute to the corrosion inhibition rate and are related to the protective properties of the coatings as a whole. The clear decrease in Q_1_ and Q_2_ magnitudes is obvious for the sample obtained in electrolyte with a concentration of halloysite nanotubes of 20 g/L due to the H20 sample’s optimal combination of porosity and sublayer thickness. 

Table 6 shows an overall reduction in the anti-corrosive properties of the samples after exposure to the corrosive medium for 24 h. This result is a consequence of the corrosive medium reaching the substrate through the defects in the coating. The order in the set of the studied samples remain unchanged: the H20 sample possesses the highest |Z|_f = 0.01 Hz_.

**Figure 13:**
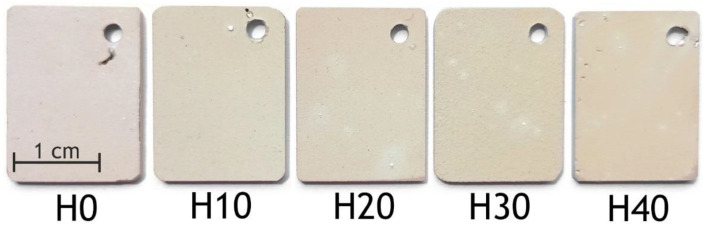
The appearance of H0, H10, H20, H30, H40 samples after 28 days of exposure to 3.5 wt.% NaCl solution.

The protective properties of the coatings were also tested by a 28-day immersion in 3.5 wt.% NaCl. The least number of defects was found on the H20 and H30 samples, while for the base PEO coating, pitting corrosion was observed (Figure 13). 

### 2.3. Mechanical Properties of the Coatings

Beyond the chemical resistance, silicon and aluminum oxides are expected to improve the mechanical properties of the coatings, which were assessed using a DUH–W201 tester for the calculation of microhardness and Young’s modulus (Table 7). The coatings formed in the electrolytes containing 10 and 20 g/L of HNTs demonstrated the highest microhardness and Young’s modulus among all samples, which is attributable to their low porosity and the presence of the HNTs dihydroxylation products.

The results presented in Table 7 illustrate that the presence of Al_2_O_3_ enhances the microhardness of the coatings containing HNTs in comparison with the base PEO coating by 1.3–1.5 times. This parameter begins to decrease for the samples obtained in electrolytes with the addition of HNTs above 20 g/L, which apparently stems from their porosity.

**Figure 14:**
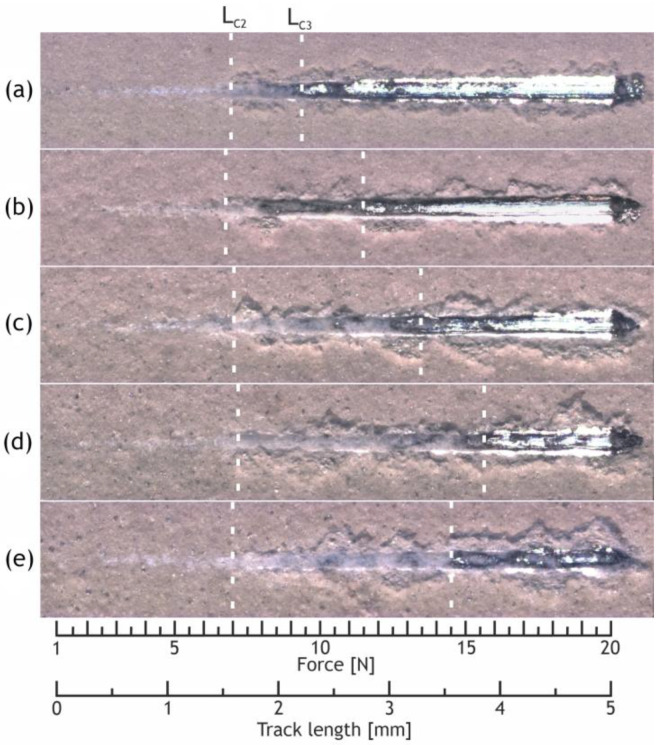
Images of scratch failures of the H0 (**a**), H10 (**b**), H20 (**c**), H30 (**d**), H40 (**e**) samples.

Figure 14 represents the images of the studied samples after the scratch testing. All samples containing nanoparticles demonstrated L_C3_ magnitudes exceeding those for the base PEO coating (Table 8). The highest L_C2_, L_C3_ parameters values were demonstrated by the H30 sample. Microhardness, thickness, and porosity factored crucially into the adhesion strength of the tested coatings; therefore, the L_C3_ parameter began to decrease for the highly heterogeneous and porous H40 sample.

### 2.4. Release Tests 

According to the results of the provided studies, the optimal mechanical and electrochemical characteristics were demonstrated by the samples obtained in the electrolyte containing 20 g/L of HNTs; therefore, release tests were carried out using the samples obtained in electrolytes with this concentration of HNTs. 

Since aluminum ions are essentially toxic to the human body [69,70,71], we conducted an experiment aimed at determining the possibility of Al^3+^ release from the coatings. The samples were immersed in a solution imitating human blood plasma by ionic composition (SBF) for 28 days, after which the solution was analyzed by atomic adsorption spectroscopy (AAS). According to the data obtained, the concentration of aluminum ions in the solution is below the detection limit of the AAS method.

The H20-P samples were prepared using HNTs pre-loaded with caffeine, which allowed us to estimate the applicability of the PEO process for the formation of the coatings with a sustained release of active molecules and assess the maintenance of such molecules in the loaded HNTs lumen throughout the PEO process.

The H20-E samples obtained with pristine HNTs were immersed in caffeine-containing electrolytes, washed, and tested. These samples allowed us to account for the adsorption of caffeine from the electrolyte by the sample surface and compare release rates of the pre-loaded and raw HNTs. 

The H0-O samples were obtained in a caffeine-containing electrolyte without HNTs, which allowed us to assess the adsorption capacity of the base oxide layer itself and estimate the possible seizure of the caffeine from the electrolyte by the forming oxide layer.

To assess the capability of the loading of the formed coatings containing HNTs with active molecules and their adsorptive properties, as well as the possibility of the application of such coatings for sustained release of the substances, the release tests for samples that were exposed to the caffeine solution (H20-C, H0-C) were performed. Both H20-C and H0-C were obtained in caffeine-free electrolyte and then exposed to the concentrated caffeine solution, which allowed us to compare and estimate the adsorption capacities of the samples without pretreatment.

**Figure 15:**
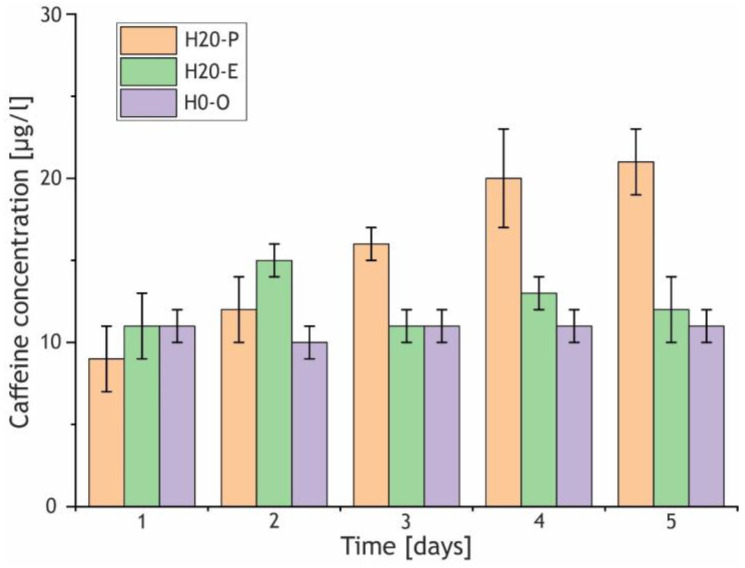
Concentration of caffeine in the release medium for the H20-P, H20-E, and H0-C samples. Data are represented as means ± SD (n = 3).

As can be seen from Figure 15, the participation of the pre-loaded HNTs in the PEO process elicits prolonged release from the H20-P sample in comparison with the H20-E and H0-C samples. The values exhibited by the H20-E and H0-C coatings correspond to a fluctuation process in the region of a certain equilibrium value of the caffeine concentration, while for the H20-P sample, the concentration of the active molecules increased with the exposure time. The dynamics of the release for coatings with caffeine-loaded HNTs favorably differs from those exhibited by the H20-E and H0-O samples and indicates that part of the caffeine remains in the nanotubes’ lumen after the PEO.

**Figure 16:**
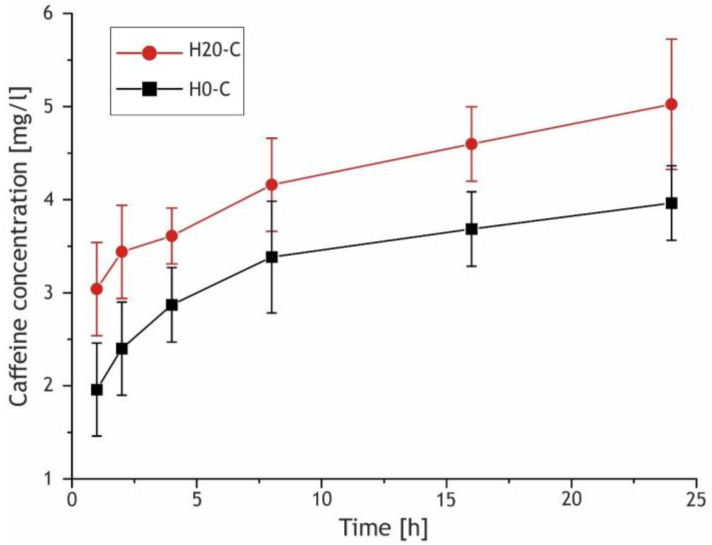
Caffeine release curves for the H20-C and H0-C samples (solid lines are for perception simplifying only).

Figure 16 demonstrates the release curves for the H20-C and H0-C samples that were exposed to the saturated caffeine solution. The samples containing HNTs exhibited higher concentrations of released caffeine compared to the H0-C, which can be explained by the adsorption activity of embedded HNTs and the more developed surface of the H20 sample, as it was noted for the SEM images and profilometric analysis of the coatings (Figure 3 and Figure 9).

### 2.5. Release Performance of the Coatings

**Figure 17:**
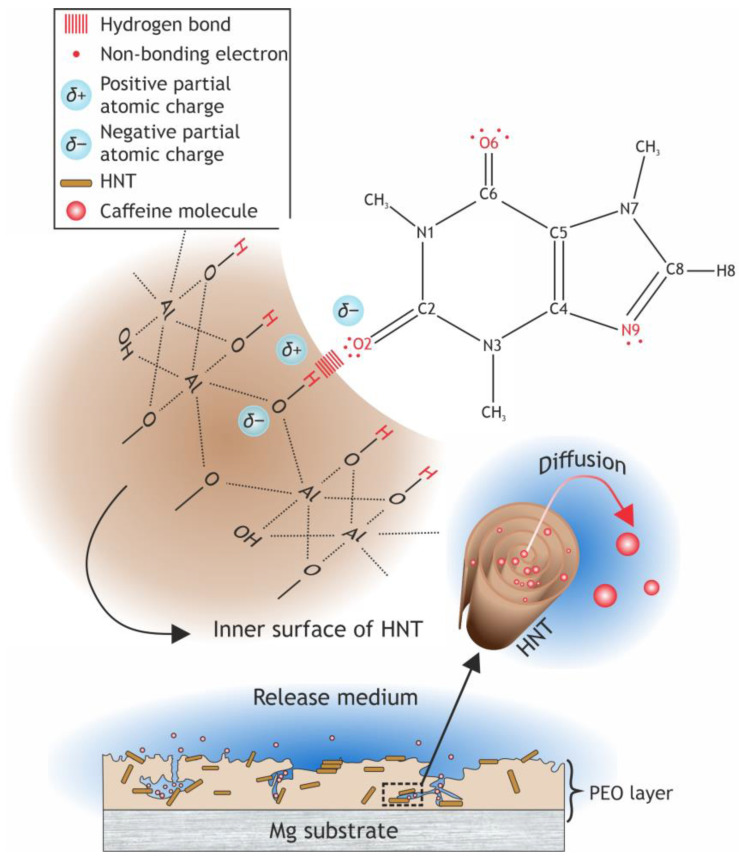
Interaction mechanism between HNTs lumen surface and caffeine.

The release of loaded organic molecules from a coating containing HNTs proceeds in two stages, including a fairly rapid desorption of molecules attached to the oxide layer and HNTs by van der Waals forces and a slower stage of the release from the inner cavity of the nanotubes [72,73,74], where they are held by hydrogen bonds (Figure 17).

Caffeine is a rather polar compound, whose molecular structure facilitates its retention in HNTs through hydrogen bonding. According to the modelling of caffeine molecule hydration, its O2, O6 and N9 atoms are prone to serve as hydrogen bond acceptors due to their partial negative charge [75]. Carbonyl moieties contain O2 and O6 atoms with two pairs of non-bonding electrons that interact electrostatically with positively charged hydrogen of hydroxy groups in HNTs lumen [49,76]. The N9 atom might provide weak hydrogen bonding with its pair of electrons as well [75]. 

The mobile structure and high dipole moment make it possible for the caffeine molecule to intercalate between the HNTs layers by breaking hydrogen bonds, dipole–dipole interactions and van der Waals forces, holding alumosilicate layers together [51]. A high degree of interlayer hydration of halloysite-10 Å (Figure S1) favors complex formation [51,77] due to expanded interlayer space, where the caffeine solution penetrates. However, complexation requires both donor and acceptor functional groups as two bonding sites [51,78,79], while caffeine has only acceptor groups. Therefore, this type of interaction is controversial and needs to be confirmed. 

Another point to consider is the electronic properties of the HNTs, in particular, the negative charge of the inner surface of nanoparticles that presumably implies some electrostatic interactions with the caffeine dipole, contributing to the adsorption capacity of the coatings besides hydrogen bonding [80].

As schematically represented in Figure 17, caffeine molecules diffuse down the concentration gradient after the hydrogen bonds are broken by thermally activated perturbations or natural fluctuations [81]. Then caffeine accumulates in sinuous channels in which seized HNTs are located. A complex structure of channels retards the fast penetration of the release medium and decelerates the ingress of caffeine into the bulk of the medium, which supports a sustained release. Apparently, the phenomenon of the prolonged release exhibited by the H20-P samples can be attributed to the gradual release of caffeine from channels of the coating, where loaded HNTs are distributed (Figure 17), whereas equilibrium concentrations of caffeine for the H20-E and H0-C are reached rapidly due to the desorption of the intercalating agent from the coatings surface. 

Moreover, the H20-C and H20-P coatings containing the HNTs demonstrate higher adsorption capacity due to hydrogen bonding with active molecules, compared to the H0-C and H0-O that adsorb caffeine by the weaker van der Waals forces with their oxide layer.

## 3. Materials and Methods 

### 3.1. Samples Preparation

The rectangular specimens of 20 mm × 15 mm × 2 mm in size made of Mg-Mn-Ce magnesium alloy (Mn 1.30; Ce 0.15; Mg bal. (wt.%)) were used as a substrate. The specimens were mechanically ground with a sanding paper of various grits (P600, P800, and P1200), cleaned in an ultrasonic bath Sonorex RK100H (Bandelin, Germany) filled with deionized water. Then samples were degreased with isopropanol and air-dried. 

### 3.2. Coatings Formation

Based on the positive results of previous studies [21,82], the solution containing sodium fluoride (5 g/L) and sodium silicate (20 g/L) was chosen as the base electrolyte. The conductivity of this electrolyte was equal to 16–17 mS/cm, and the pH was equal to 10.7–10.8.

In this work, we used halloysite nanotubes (Halloysite Ural, Russia) with a length of 1–3 μm, an outer diameter of 50–70 nm and a lumen diameter of 15–30 nm (Figure S2). The nanoparticles were dispersed in the base electrolyte using a Sonopulse HD 3200 ultrasonic homogenizer (Bandelin, Germany). 

An anionic surfactant (NaC_12_H_25_SO_4_, sodium dodecyl sulfate) was used for stabilization and intensification of the electrophoretic migration of the HNTs dispersed phase. The surfactant concentration in the electrolyte was 0.25 g/L. The concentration of the HNTs in the prepared electrolyte was 0, 10, 20, 30, and 40 g/L (Table 9). Reagent grade chemicals were used in this research.

The process of coatings formation was carried out using the plasma electrolytic oxidation unit. The methodology of the PEO process was described elsewhere [21]. During the PEO, the polarizing pulse frequency was equal to 300 Hz. All samples were processed in the two-stage bipolar PEO mode. During the first stage (200 s), the anodic and cathodic components were in galvanostatic (0.36 A/cm^2^) and potentiostatic (−30 V) modes, respectively. For the second stage (600 s), the anodic component remained galvanostatic (0.36 A/cm^2^), while the cathodic one changed potentiodynamically from −30 V up to −10 V. The electrolyte temperature was maintained at 10 °C by a recirculating water chiller Smart H150-3000 (LabTech, Italy).

### 3.3. Morphology and Composition Characterization

The study of the surface topography was conducted by the optical laser profilometry method using an OSP370 device installed on an M370 workstation (Princeton Applied Research, TN, Oak Ridge, USA). Image analysis was performed using Gwyddion 2.45 software. The surface topography was characterized by the most common roughness parameters: R_a_ (arithmetical mean deviation of the profile), and R_z_ (ten-point height of irregularities).

The microphotographs of the surface of the samples were obtained using a Sigma 300 scanning electron microscope (SEM) (Carl Zeiss, Munich, Germany). The elemental composition of the surface layers was determined by energy dispersive spectroscopy (EDS) using an INCA X-act EDS analyzer integrated into the SEM (Oxford Instruments, MA, Concord, USA) and an energy dispersive X-ray fluorescence spectrometer EDX-800HS (Shimadzu, Kyoto, Japan). 

The coatings thickness was measured using SEM images of the samples cross-section.

The porosity (P) was determined by digital processing of the SEM images using ImageJ software (National Institutes of Health, MD, Bethesda, USA). The proportion of the area occupied by pores on the entire visible surface of the coating was estimated according to Equation (1):

(2) $$P=\frac{\sum_{j}{\left(S_{P}\right)}_{i}}{S_{0}}\cdot 100\%,$$

where (*S_p_*)*_i_* is the *i*-th pore area, and S_0_ is the area of the analyzed surface.

X-ray photoelectron spectroscopy (XPS) was used for the analysis of the chemical composition of the investigated coatings. The XPS measurements were carried out using a SPECS device (SPECS, Germany) with a 150 mm hemispherical electrostatic analyzer. Ionization was carried out with non-monochromatized Al K_α_ radiation. The transmission energy of the analyzer was 50 eV. The step was 0.1 eV for high resolution spectra and 1 eV for survey spectra. The scale was calibrated using the peaks of C 1 s hydrocarbons (E_b_ = 285.0 eV). An Ar^+^ ions source with an E_k_ of 5000 eV was used to etch the samples; the etching time was equal to 5 min, and the average etching rate was equal to 10 Å/s.

The X-ray diffraction (XRD) technique was used for the phase analysis of the HNTs and of the obtained coatings. XRD was performed on a Bruker D8 ADVANCE diffractometer (Bruker). The diffraction patterns were recorded in a 4–80 degrees (2θ) range using a monochromatic Cu K_α_ radiation with a step size of 0.02° and speed of 1 s per step, operating at 40 kV at 40 mA. 

### 3.4. Electrochemical Measurments 

The electrochemical tests were carried out using VersaSTAT MC (Princeton Applied Research, TN, Oak Ridge, USA). Potentiodynamic polarization was performed at room temperature using three-electrode K0235 FlatCell (Princeton Applied Research, TN, Oak Ridge, USA). The samples were studied in 3.5 wt.% NaCl solution. The counter electrode was a platinized niobium mesh. The saturated calomel electrode (SCE) was used as a reference electrode. The area of contact of the sample with the electrolyte was equal to 1 cm^2^. For potentiodynamic polarization tests, samples were immersed in the electrolyte for 60 min to stabilize the electrode potential. The sweep rate for potentiodynamic polarization was equal to 1 mV/s. The samples were polarized from E_corr_ − 0.15 V up to E_corr_ + 0.5 V, where E_corr_ is the corrosion potential.

Values of the corrosion potential (E_corr_), corrosion current density (*i_corr_*) and the cathodic and anodic Tafel slopes (β_c_ and β_a_, respectively) for the samples were calculated using the Levenberg–Marquardt (LEV) approach using Equation (2):

(3) $$I=i_{corr}\left({10\;}^{\left(\;\frac{{E-E}_{\mathrm{corr}}}{\beta_{a}}\right)}{+10}^{-\left(\;\frac{{E-E}_{\mathrm{corr}}}{\beta_{c}}\;\right)}\right),$$

The polarization resistance (R_P_) was calculated in accordance with Equation (3). The specimens were polarized from E_corr_ − 0.02 V up to E_corr_ + 0.02 V at sweep rate of 0.167 mV/s:

(4) $$R_{p}={\left(\frac{\Delta E}{\Delta I}\right)}_{\Delta E\to 0}\;,$$

The electrochemical impedance spectroscopy (EIS) test was carried out in a frequency range from 1 MHz to 10 mHz, using a 10 mV amplitude sinusoidal voltage. EIS measurements were conducted after 2- and 24 h exposures to the corrosive media (3.5 wt.% NaCl). Impedance spectra were acquired at a logarithmic sweep of 10 points per decade. EIS spectra were fitted using appropriate equivalent electrical circuits. 

The laboratory-based immersion corrosion test was carried out by immersing the obtained samples in a 3.5 wt.% NaCl solution for 28 days, followed by a visual inspection.

### 3.5. Mechanical Properties Characterization

The study of mechanical properties (the microhardness and elasticity modulus) was carried out using a DUH–W201 dynamic ultra-micro hardness tester (Shimadzu, Japan). The universal microhardness H_μ_ was measured on the samples cross-section using a Berkovich indenter at a load of 100 mN.

The adhesive properties of the surface layers were investigated by scratch testing using a Revetest Scratch Tester (CSM Instruments, Switzerland). A Rockwell diamond indenter was used for scratch testing. The experiments were carried out at a track length of 5 mm with a gradual increase of the applied load from 1 to 20 N at a rate of 9.5 N/min. The following parameters were determined for each coating: L_C2_ is the load at which the beginning of peeling of coating areas was observed, and L_C3_ is the load at which abrasion of the coating to the substrate occurs.

### 3.6. Release Tests

An effective adsorption of organic molecules by HNTs’ interlayer depends on the dipole moment of the intercalating agent [83,84,85]. Therefore, caffeine was chosen, due to its relatively high dipole moment [86] and the feasibility of its minor amount determination by high-performance liquid chromatography (HPLC). Caffeine was purchased from Sigma-Aldrich (99%). The HPLC system consisted of a Shimadzu LC-20AD HPLC pump, a Shim-pack FLC-ODS column, a Shimadzu SPD-M20A detector (all Shimadzu, Japan), and a water/acetonitrile (80/20) mobile phase was used at flow rate of 0.4 mL/min.

The adsorption capacity was assessed through a comparison of the release rates of caffeine from coatings that were loaded by different procedures described in Table 10. The PEO process parameters were the same as previously described for all samples used in the release tests.

The H20-P coatings were obtained in the electrolyte containing 20 g/L of the HNTs pre-loaded with caffeine. The loading of the HNTs was performed in a saturated water solution of caffeine (2.1 g of caffeine per 100 mL deionized water). A total of 20 g of the nanoparticles powder was suspended in 100 mL of the caffeine solution and kept under 100 Pa pressure for 10 min using an Epovac vacuum impregnator (Struers, Germany) [87]. The cyclic vacuum treatment was repeated three times. The suspension was then left under atmospheric pressure for 48 h at room temperature (25 °C) and continuous steering. After the exposure, HNTs were separated from the solution by decantation, followed by filtration. The excess caffeine was washed off with 100 mL of deionized water. To prevent an unintended release of caffeine from HNTs cavity during the washing, cold water (10 °C) was used. The caffeine-loaded nanoparticles were dried at 25 °C and then used in the PEO process.

The H20-E samples were obtained by exposure of the H20 samples to the base electrolyte for PEO with the addition of caffeine of 7 g/L. The concentration of the caffeine was chosen due to the proximity of the saturation point at temperature (10 °C) [88]. The exposure time was equivalent to the PEO process duration (800 s). 

The H0-C coatings were obtained in the base electrolyte, which contained 7 g/L of caffeine. 

According to several studies, HNTs’ release rates are naturally high and the release pinnacle locates within 3–4 days [33,35,37], therefore one of the release tests lasted for 5 days. Each of the studied samples were placed in tubes filled with 20 mL of deionized water and sustained at 37 °C, concentration of the caffeine in the release medium was measured every day by HPLC. 

In a separate 24 h experiment, the H0 and H20 samples were immersed in the concentrated solution of caffeine and then tested. The samples were placed for 1 h in the 100 mL water containing 2.1 g of caffeine, which is close to its saturation point (at 25 °C) [88]. After the exposure, the samples were washed with cold deionized water (10 °C). Then the samples were placed in tubes filled with 20 mL of deionized water and maintained at 37 °C in a circulation thermostat for 1 h. After 1 h, the samples were washed with deionized water and placed in another tube for 2 h, and then repeated in a similar way for 4, 8, 16, and 24 h. The concentration of caffeine in the release medium was measured by HPLC.

The data were considered to be significantly different at *p* < 0.05. The release test data are presented as mean values with standard deviation (mean ± SD).

## 4. Conclusions

The influence of HNTs on the PEO coatings structure, mechanical properties, and electrochemical properties was investigated in the present work. The increase in porosity and heterogeneity of the formed layers is directly proportional to the concentration of the HNTs in the electrolyte. The composition analyses showed that the obtained coatings include not only the HNTs, but also the products of their dihydroxylation under the plasma discharge conditions. The HNTs and products of their plasma–chemical reactions factor into the mechanical and anticorrosive properties of the PEO coatings.

The 2-fold decrease in corrosion current density was observed for the samples obtained in the electrolyte with the HNTs concentration of 20 g/L in comparison with the base PEO coating. These coatings also demonstrate the highest polarization resistance and impedance modulus after both 2 and 24 h of immersion in the corrosive medium. The achieved results can be explained by the non-porous sublayer thickness of the H20 sample and the filling of the pores with the chemically stable nanoparticles along with the products of their thermochemical transformation. Moreover, H20 and H30 samples have the highest microhardness values, which are 1.5 times higher than the base PEO layer. Results of our analysis allow us to conclude that aluminum oxide presence has a major contribution to the increase in protective properties of the coatings. 

It was found that coatings with nanocontainers that exhibit sustained release of the organic molecules can be obtained by the incorporation of both pristine and pre-loaded halloysite nanotubes during the PEO. 

The pre-loaded HNTs retain guest molecules in their lumen throughout the PEO and actuate the release of the hosted substance from the coatings with such nanocontainers. As it was shown in the research, these coatings demonstrate at least a 5-day-long release of caffeine. Furthermore, coatings with pristine HNTs display higher concentrations of the released caffeine, in comparison with the base PEO layer. In both cases, the higher adsorption of caffeine and sustained release are presumably attributable to the preservation of caffeine in the HNTs lumen assisted by hydrogen bonds. 

The obtained results lead us to conclude that HNTs remain their shape and adsorptive properties after the incorporation into the PEO coatings, which provides the opportunity for the one-step formation of protective coatings on magnesium alloys with an active molecule-delivery property.

## Figures and Tables

**Table 1:** Thickness and roughness parameters of the studied samples.

Sample	R_a_ [µm]	R_z_ [µm]	Thickness [μm]	Porosity [%]
H0	1.7 ± 0.4	10 ± 2	51 ± 5	5.4 ± 0.3
H10	2.0 ± 0.3	11 ± 3	53 ± 6	5.7 ± 0.2
H20	2.5 ± 0.6	12 ± 3	55 ± 5	8.2 ± 0.2
H30	3.0 ± 0.7	16 ± 3	59 ± 7	9.4 ± 0.2
H40	3.4 ± 0.7	17 ± 3	62 ± 8	10.9 ± 0.3

**Table 2:** The elemental composition of the studied coatings.

Sample	Content of Elements [at. %]						
	O	Si	Al	Mg	Na	Mn	Other
H0	52.8	13.3	–	29.3	2.7	0.9	1.0
H10	45.1	17.4	7.2	26.4	2.6	0.9	0.4
H20	46.2	19.5	10.8	19.7	2.5	0.8	0.5
H30	46.6	20.5	12.6	15.9	3.0	0.7	0.7
H40	47.3	21.9	14.8	11.5	2.7	0.7	1.1

**Table 3:** Binding energy [eV] and the concentration of the main elements [at. %] for the raw halloysite nanotubes powder and H40 sample.

Sample	Content of Elements [at. %]						
	Na (1s)	F (1s)	O (1s)	C (1s)	Si (2p)	Al (2p)	Mg (2p)
Raw powder	–	685.0 (1.5)	532.7	285.0 (4.8)	103.7	75.3 (12.9)	–
			531.7		100.8		
			(62.8)		(18.0)		
H40 as-prepared	1071.2 (11.3)	684.5 (3.8)	532.2	289.7 286.7 285.0 (4.6)	103.1 101.3 (18.0)	74.9 (5.1)	50.9 (4.2)
			530.9				
			(53.0)				
H40 after Ar^+^ etching	1071.2 (5.5)	684.5 (2.6)	532.2 530.9 (56.3)	289.7	103.1 101.2 (21.5)	74.9 (6.9)	51.0 (6.5)
				286.7			
				285.5			
				283.3			
				(0.9)			
Possible chemical state	Na^+^	F^−^	SiO_2_ MeO_x_	OCO	Si^4+^ Si^x+^	Al^3+^	MgO
				COC			
				CC			
				MeCx			

**Table 4:** Corrosion properties of studied samples.

Sample	R_p_ [Ohms∙cm^2^]	i_corr_ [Amp/cm^2^]	E_corr_ [V]	β_a_ [mV/decade]	β_c_ [mV/decade]
H0	6.5 × 10^5^	1.1 × 10^−7^	−1.46	773	237
H10	8.6 × 10^5^	5.7 × 10^−8^	−1.50	1636	152
H20	1.2 × 10^6^	4.9 × 10^−8^	−1.45	864	269
H30	1.1 × 10^6^	5.6 × 10^−8^	−1.47	1070	184
H40	2.9 × 10^5^	1.0 × 10^−7^	−1.52	1046	170

**Table 5:** Calculated parameters of equivalent electrical circuits (the units of R and |Z|_f = 0.01 Hz_ are Ω × cm^2^; Q are S × cm^−2^ × s^n^) for the coatings after 2 h immersion in 3.5 wt.% NaCl aqueous solution. |Z|_f = 0.01 Hz_ was measured at the frequency f = 0.01 Hz.

Sample	|Z|_f = 0.01 Hz_	R_1_	CPE_1_		R_2_	CPE_2_	
			Q_1_	n		Q_2_	n
H0	1.21 × 10^5^	2.23 × 10^4^	3.4 × 10^−7^	0.79	1.06 × 10^5^	6.45 × 10^−8^	0.92
H10	4.61 × 10^5^	3.57 × 10^4^	1.80 × 10^−7^	0.77	1.56 × 10^5^	7.36 × 10^−8^	0.91
H20	1.26 × 10^6^	1.72 × 10^5^	1.12 × 10^−7^	0.82	1.30 × 10^6^	6.51 × 10^−9^	0.95
H30	7.02 × 10^5^	1.20 × 10^5^	2.20 × 10^−7^	0.82	6.28 × 10^5^	5.97 × 10^−7^	0.86
H40	2.61 × 10^5^	3.36 × 10^4^	3.10 × 10^−7^	0.80	2.76 × 10^5^	9.04 × 10^−7^	0.71

**Table 6:** Calculated parameters of equivalent electrical circuits (the units of R and |Z|_f = 0.01 Hz_ are Ω × cm^2^; Q are S × cm^−2^ × s^n^) for the coatings after 24 h immersion in 3.5 wt.% NaCl aqueous solution. |Z|_f = 0.01 Hz_ was measured at the frequency f = 0.01 Hz.

Sample	|Z|_f = 0.01 Hz_	R_1_	CPE_1_		R_2_	CPE_2_	
			Q_1_	n		Q_2_	n
H0	5.96 × 10^4^	1.20 × 10^3^	4.11 × 10^−7^	0.72	6.95 × 10^4^	4.00 × 10^−7^	0.84
H10	1.18 × 10^5^	9.53 × 10^3^	1.15 × 10^−7^	0.79	1.17 × 10^5^	1.09 × 10^−7^	0.95
H20	9.94 × 10^5^	8.27 × 10^3^	5.47 × 10^−7^	0.71	1.05 × 10^6^	6.36 × 10^−8^	0.95
H30	1.52 × 10^5^	1.73 × 10^4^	5.27 × 10^−7^	0.78	1.92 × 10^5^	2.29 × 10^−8^	0.98
H40	1.20 × 10^5^	1.39 × 10^5^	1.06 × 10^−6^	0.83	–	–	–

**Table 7:** Microhardness and Young’s modulus of the obtained coatings.

Sample	H_μ_ [GPa]	Young’s Modulus [GPa]
H0	4.5 ± 0.4	76 ± 10
H10	7.3 ± 0.3	139 ± 6
H20	7.3 ± 0.5	143 ± 10
H30	6.3 ± 0.9	118 ± 11
H40	5.7 ± 0.6	107 ± 9

**Table 8:** Critical loads for the studied coatings, determined by the scratch test.

Sample	L_C2_ [N]	L_C3_ [N]
H0	6.9 ± 0.2	9.4 ± 0.6
H10	6.7 ± 0.1	11.5 ± 0.4
H20	6.9 ± 0.1	13.5 ± 0.8
H30	7.2 ± 0.1	15.6 ± 0.4
H40	7.0 ± 0.5	14.5 ± 0.3

**Table 9:** Components of the used electrolytes.

Sample	The Concentration of the Electrolyte Component [g/L]			
	Na_2_SiO_3_∙5H_2_O	NaF	NaC_12_H_25_SO_4_	HNTs
H0	20	5	0	0
H10			0.25	10
H20				20
H30				30
H40				40

**Table 10:** Designations of the samples for the release behavior studies.

Method of Loading with Caffeine	Sample Designation	Concentration of Caffeine in the pre-Filling Solution [g/100 mL]	Concentration of Caffeine in the PEO Electrolyte [g/100 mL]
5 days test			
H20 samples obtained with HNTs that were pre-loaded in the caffeine solution	H20-P	2.1	–
H20 samples exposed to the caffeine containing electrolyte	H20-E	–	0.7
H0 samples obtained in the caffeine-containing electrolyte	H0-O	–	0.7
24 h test			
H20 sample exposed to the caffeine solution	H20-C	2.1	–
H0 sample exposed to the caffeine solution	H0-C		

## Data Availability

The data presented in this study are available on request from the corresponding author. The data are not publicly available due to privacy reasons.

## Supplementary Materials

The following supporting information can be downloaded at: https://www.mdpi.com/article/10.3390/ijms24010305/s1, Figure S1: X-ray diffraction pattern of the used halloysite nanotubes. Figure S2: SEM image of the used halloysite nanotubes.
