# Supplementary material for: PEO Coatings Modified with Halloysite Nanotubes: Composition, Properties, and Release Performance
Source: Int J Mol Sci. 2022 Dec 24;24(1):305. doi: 10.3390/ijms24010305 (PMC9820610; doi:10.3390/ijms24010305)
Supplement: Supplementary file 1 [file ijms-24-00305-s001.zip › ijms-2095701-supplementary.pdf]

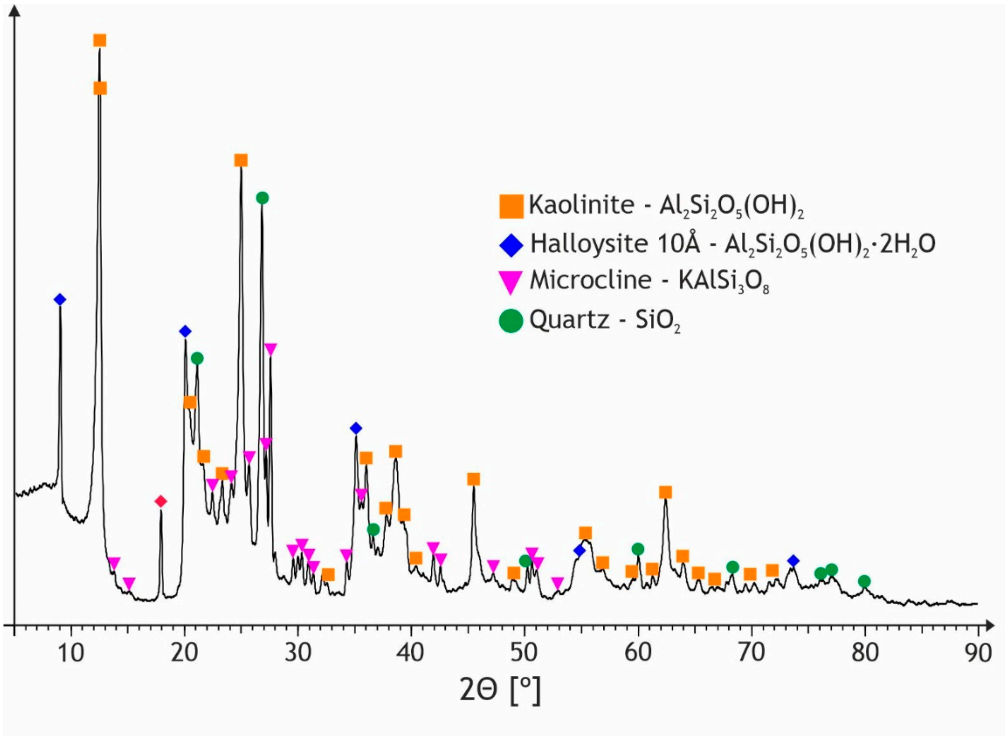

Figure. S1. X-ray diffraction pattern of the used halloysite nanotubes.

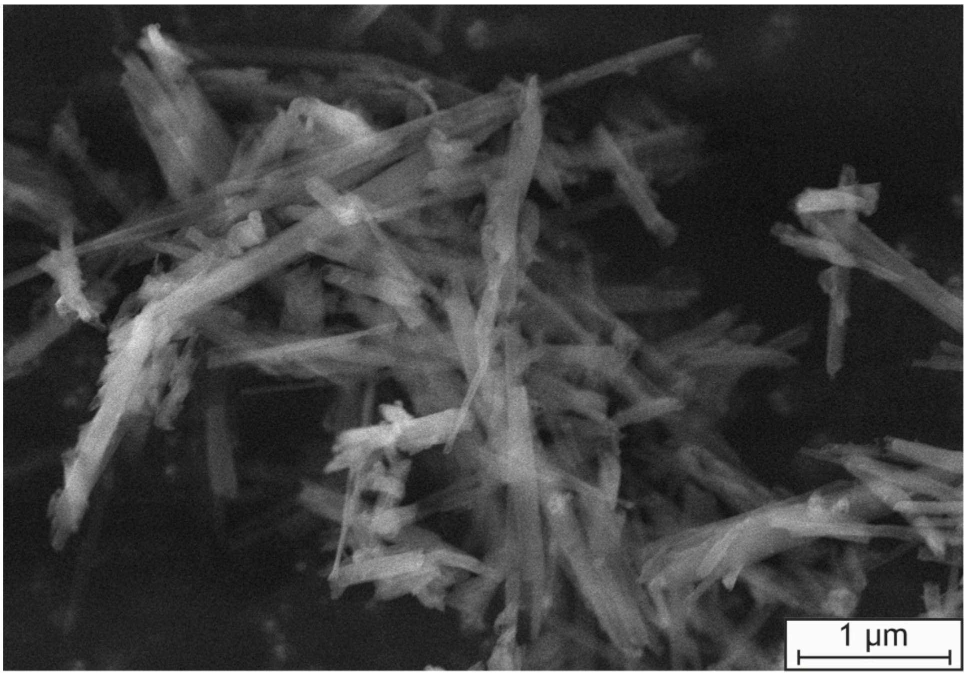

Figure. S2. SEM image the used halloysite nanotubes.
